# Supplementary material for: Location patterns and longitudinal progression of white matter hyperintensities
Source: medRxiv. 2026 Feb 23:2026.02.20.26346709. Preprint. [Version 1] doi: 10.64898/2026.02.20.26346709 (PMC12970357; doi:10.64898/2026.02.20.26346709)
Supplement: Supplement 1 [file media-1.pdf]

Supplementary materials

e1 Methods

e1.1 Datasets

The multi-cohort dataset included ADNI3 scans collected between 2017 and 2022, Insight46 scans collected between 2015 and 2019, SABRE scans collected between 2014 and 2019, UKB scans since 2014, and OASIS-3 collected over the course of 30 years.

ADNI3 ADNI3 is a multi-centre, natural history, prospective study based in North America [18], [19], [20]. Data collection for ADNI3 began in 2016, and was expected to conclude by the end of 2024. Participants were aged 55-90 at baseline, with no significant neurological disease other than Alzheimer's disease. In addition to MRI scans, a comprehensive set of demographic information (*e.g.* age, sex), medical history (*e.g.* systolic and diastolic blood pressures), neuropsychology tests (*e.g.* ADAS11/13, MMSE), positron emission tomography (PET) and genotype data (*e.g.* APOE4 genotype) are also available.

Insight46 Insight46 is the prospective neuroimaging substudy of the MRC National Survey for Health and Disease (NSHD) birth cohort [21], [22]. It drew a subset of 502 participants from this cohort of individuals all born in the same week of March 1946. Baseline neuroimaging data were collected between 2015 and 2017, when participants were aged 69-71, and follow-up data were collected between 2018 and 2020, when participants were aged 73-75. The study collected clinical, neuropsychological, brain MRI scans, PET amyloid imaging, blood, urine and CSF biomarkers.

SABRE The SABRE (Southall and Brent Revisited) cohort study is a longitudinal project based in West London [23]. It originally focused on cardiovascular disease and diabetes in people of White British, first-generation migrants of South Asian or African Caribbean heritage. Baseline data were collected between 1988 and 1991 (when participants were aged between 40 and 69). At the third wave of data collection (2014-2019), neuroimaging data were acquired for 772 participants and their spouses on a 3T Philips Achieva Scanner.

UKB UK Biobank recruited approximately 500,000 participants between 2006 and 2010 [24]. Data collected include demographic, lifestyle, clinical, biochemical, genetic and imaging measures, with linkage to electronic health records. A subset of approximately 67,000 participants underwent brain MRI scans [25], with follow-ups available for some participants.

Validation dataset: OASIS-3 OASIS-3 is a longitudinal neuroimaging, clinical, cognitive, and biomarker dataset that retrospectively collected data from 1378 participants in the United States aged between 42 and 95 [26]. It includes participants who were cognitively normal as well as those at various stages of cognitive impairment and dementia. Data collection was conducted at a single site at the Knight Alzheimer Disease Research Center at Washington University in St. Louis, Missouri.

e1.2 Quality control

Following segmentation, we first conducted a visual inspection of WMH masks from ten ADNI3 scans. This was followed by further inspection of scans from both UKB and ADNI3 datasets,

focusing on suspected anomalies - specifically, those with either a high volume of WMH lesions (over 80mL) or an absence of WMH lesions. Any incorrect or erroneous records were either re-processed (ADNI3: n=12 at baseline, UKB: 4 at follow-up) or excluded (ADNI3: n=1 at baseline, n=2 at follow-up, UKB: n=16 at follow-up). Flowcharts for participants of each cohort are provided in e1.6.

e1.3 MRI protocol

eTable 1 MRI acquisition parameters across cohorts.

Cohort	Scanner vendor / model	Site	Field strength	Sequence	TR (ms)	TE (ms)	TI (ms)	Voxel size (mm^3)
**ADNI3**	GE, Siemens, Philips	Multi	3T	T1	2300	min full echo	900	1×1×1
				T2-FLAIR	4800	119	1650	1.2×1×1
**Insight46**	Biograph mMR 3T PET/MRI	Single	3T	T1	2000	2.92	870	1.1×1.1×1.1
				T2-FLAIR	5000	402	1800	1.1×1.1×1.1
**SABRE**	Philips Achieva	Single	3T	T1	6.9	3.1	-	1×1×1
				T2-FLAIR	4800	125	1650	1×1×1
**OASIS-3**	Siemens (various models)	Single	3T, some 1.5T	T1	2400	3.16	1000	1×1×1*
				T2-FLAIR	900	91	2500	1×1×1*
**UK Biobank**	Siemens Skyra	Multi	3T	T1	2000	-	880	1×1×1
				T2-FLAIR	5000	-	1800	1.05×1×1

** Note: Lower resolutions also present.*

e1.4 Availability of variables

eTable 2 Availability of non-imaging variables across cohorts. ✓indicates that the variable is available.

VARIABLE	ADNI-3	INSIGHT46	SABRE	UKB	OASIS-3
Age	✓	✓	✓	✓	✓
Sex	✓	✓	✓	✓	✓
Ethnicity	✓		✓	✓	✓
Total WMH volume	✓	✓	✓	✓	✓
APOE4 genotype	✓	✓	✓	✓	✓
Smoking status		✓	✓	✓	✓
Hypertension	✓	✓	✓	✓	✓
Anti-hypertensive medication		✓	✓	✓	✓
Systolic blood pressure	✓	✓	✓	✓	✓
Diastolic blood pressure	✓	✓	✓	✓	✓
Type 2 diabetes		✓	✓	✓	✓
Diabetic medication		✓	✓	✓	✓
HbA1c		✓	✓	✓	
Glucose	✓	✓	✓	✓	
Lipid-lowering medication		✓	✓	✓	✓
LDL cholesterol		✓	✓	✓	
HDL cholesterol		✓	✓	✓	
Triglycerides	✓	✓	✓	✓	
White blood cell count		✓	✓	✓	
Creatinine	✓	✓	✓	✓	

e1.5 Definition of variables

Ethnicity

eTable 3 Definition of ethnicity across cohorts.

HARMONISED LABEL	ADNI3	INSIGHT46	SABRE	UKB	OASIS-3
White	White	Not available	European	1	White
Black	Black	Not available	African Caribbean	4	Black
Asian (incl. Chinese)	Asian	Not available	South Asian	3, 5	Asian
Mixed	More than one	Not available	N/A	2	more than one
Other	Unknown	Not available	Other	6	Unknown

Smoking status

eTable 4 Definition of smoking status across cohorts.

HARMONISED LABEL	ADNI3	INSIGHT46	SABRE	UKB	OASIS-3
Never smoker	Not available	Never	Never	Never	
Former smoker	Not available	Ex	Ex	Ever	(processed)
Current smoker	Not available	Current	Current	Current	

Note: ADNI3 does not have smoking status recorded near the visit used for analysis. OASIS-3 smoking status was processed from other variables.

In OASIS-3, smoking status was derived using a hierarchical classification to prioritise recent behaviour. Participants were classified as current smokers if TOBAC30=1, indicating cigarette use in the past 30 days, with this category taking priority over all others. Ex-smokers were defined as those with TOBAC30=0 and evidence of past smoking, determined by at least one of the following: TOBAC100=1 (more than 100 cigarettes smoked in lifetime), SMOKYRS> 0 (non-zero years of smoking), valid QUITSMOK data (reported age at quitting), or valid PACKSPER data (reported packs per day). This category required both non-current smoking and a positive smoking history. Never smokers were defined as participants with TOBAC30=0 and TOBAC100=0, and no smoking history. Those who met all of: SMOKYRS null or 0, no quit age (QUITSMOK null), and no reported packs per day (PACKSPER null) were considered to have no smoking history. Participants with missing or insufficient information to meet these criteria were classified as unknown.

Medication use

eTable 5 Data field names used for medication variables across cohorts.

HARMONISED LABEL	ADNI3	INSIGHT46	SABRE	UKB	OASIS-3
Lipid-lowering	n/a	vasc_cholmed_bin_i46p1	q4_lipidloweringmedsv3	field 6177 (males) and 6153 (females), field 131286 (date first reported essential primary hypertension), 131294 (date of secondary hypertension)	processed from medname
Diabetic		vasc_bplwrmed_bin_i46p1	q4_gliptinv3, q4_glitazonev3, q4_glp1v3, q4_insulinv3, q4_metforminv3, q4_sureav3		
Anti-hypertensive		vasc_diabeticmed_i46p1	q4_antihypertensiveany_medsv3, q3_10b_highbpcurrentmedsv3		

e1.6 Clustering implementation

The R implementation of the stability estimation method is publicly available at https://github.com/hyu-ub/bootcluster [44]. We translated the relevant code into Python and made several modifications to suit the requirements of the analysis. Key modifications included: 1) Enabled additional clustering methods beyond k-means, as used in the original study, 2) Added outputs of cluster-wise stabilities, in addition to the original global and observational-level stabilities, 3) Incorporated functionality to reuse stability profiles for downstream tasks, and streamline experimental testing.

e1.7 5-fold cross-validation in clustering

We performed internal validation to select the optimal k. The dataset was divided into five folds, analogous to a 5-fold cross-validation procedure in classification tasks. For each iteration, stability estimation was performed at the optimal k (as well as the optimal clustering method) on the four training folds and fit to test fold before evaluation. We assumed the clustering on the entire internal dataset to be the ground truth, and map the training and original cluster assignments to each other for comparison.

e1.8 Longitudinal prediction

Uncertainty estimation of prediction models Training performance was summarised across five cross-validation folds. Balanced accuracy was summarised as the mean and ±2 standard deviations. Test and OASIS-3 performance uncertainty was estimated using 1,000 non-parametric bootstrap iterations, from which the mean and 95% confidence interval (2.5-97.5 percentiles) of balanced accuracy were derived.

e2 Results

e2.1 Participant flowchart

eFigure 1 Flowchart for ADNI3 participants

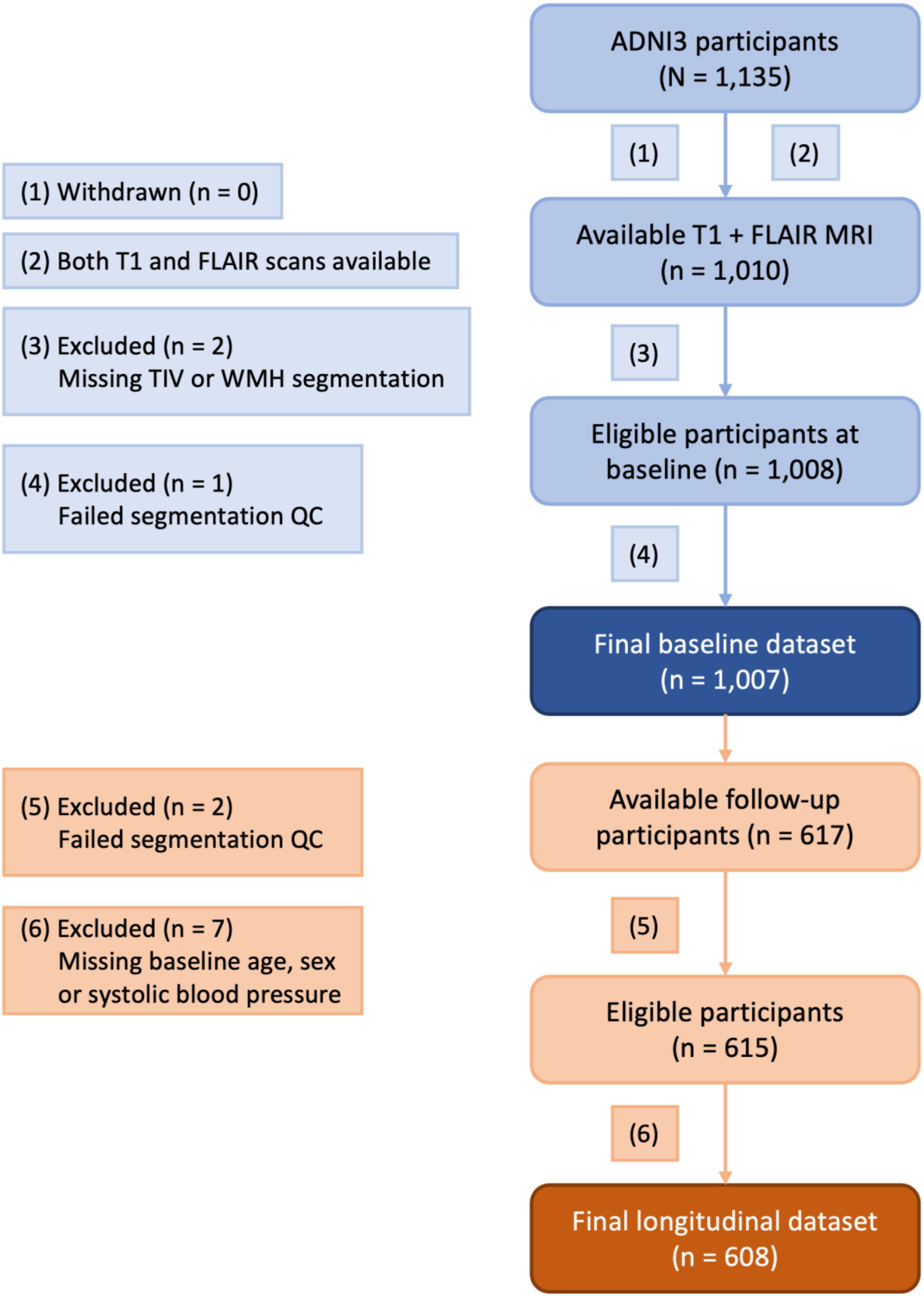

eFigure 2 Flowchart for Insight46 participants

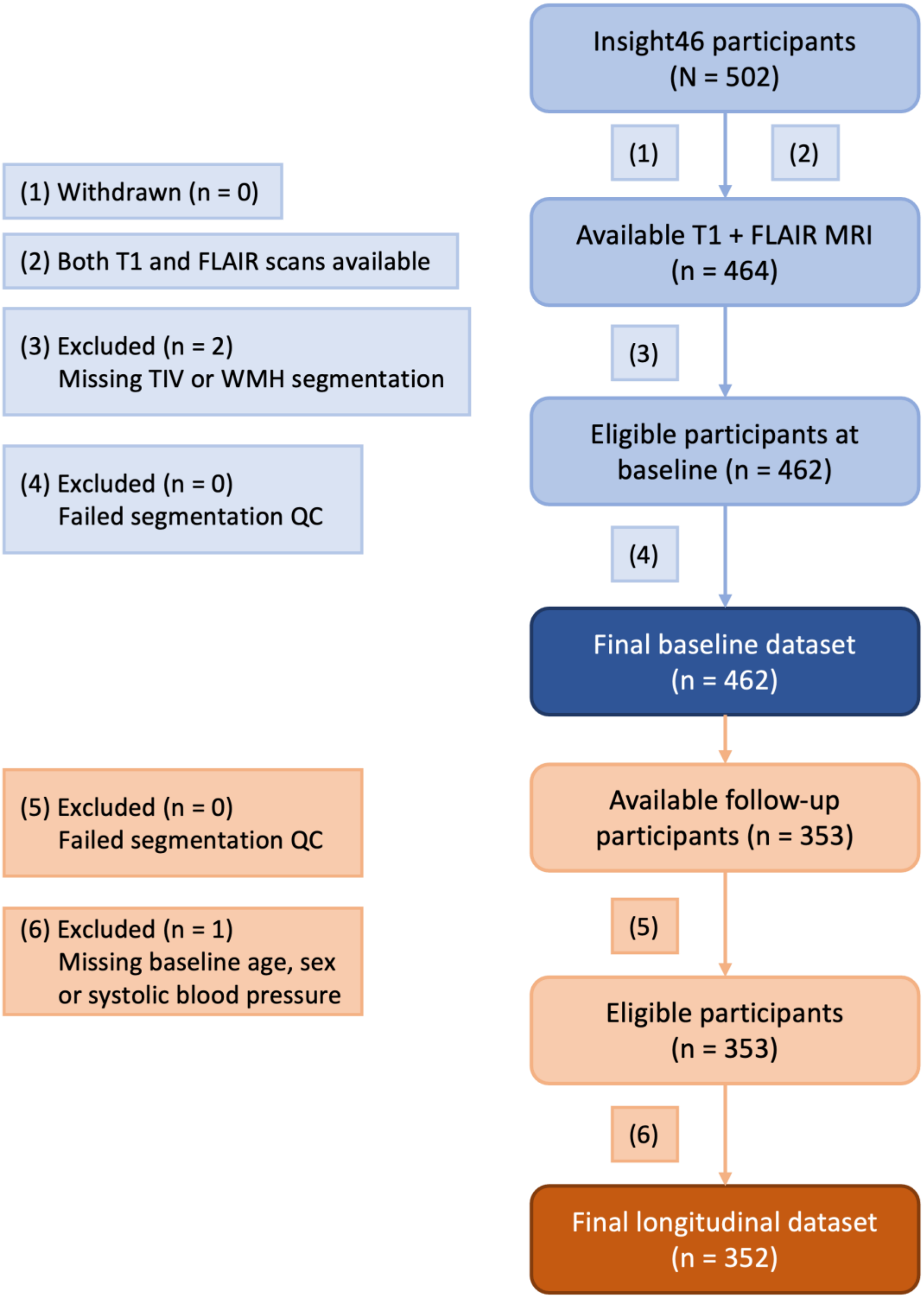

eFigure 3 Flowchart for SABRE participants

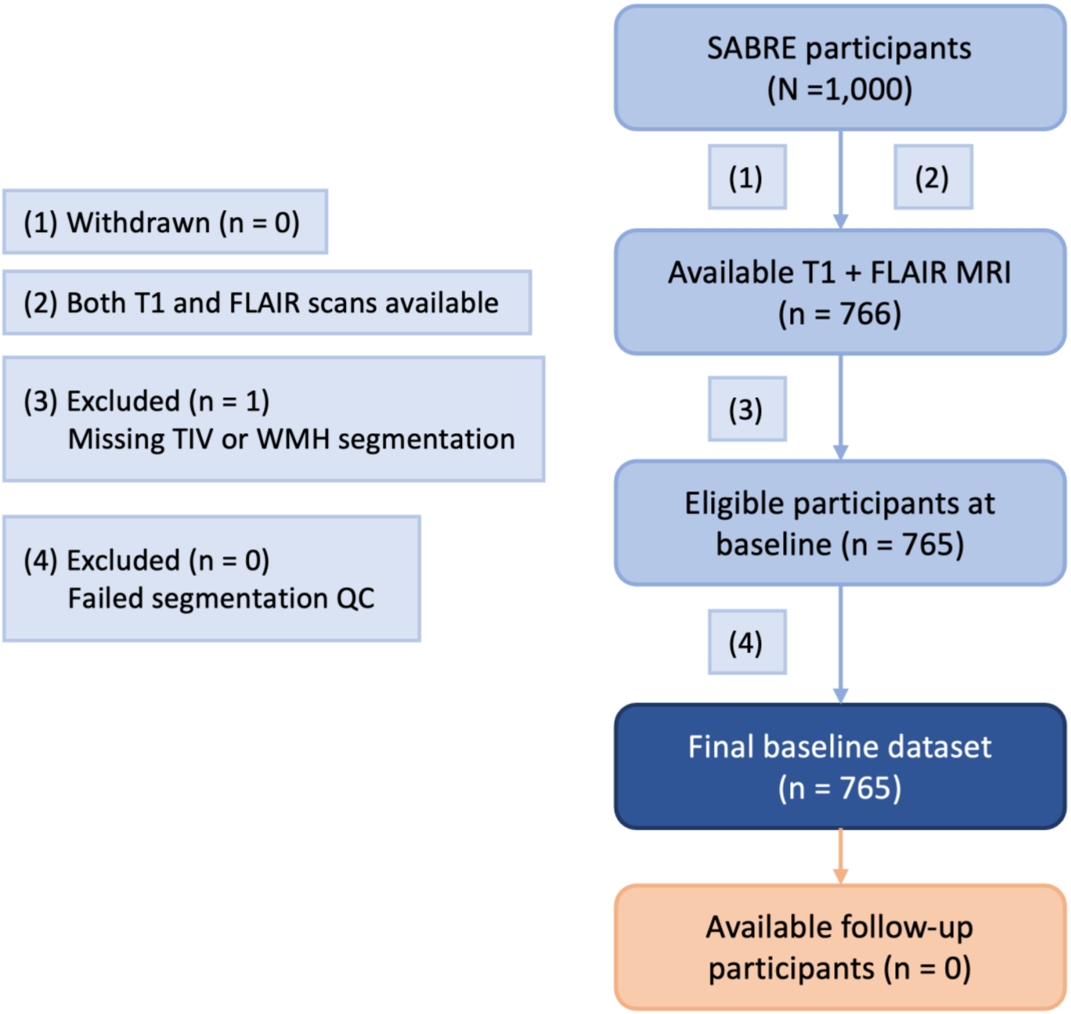

eFigure 4 Flowchart for UKB participants

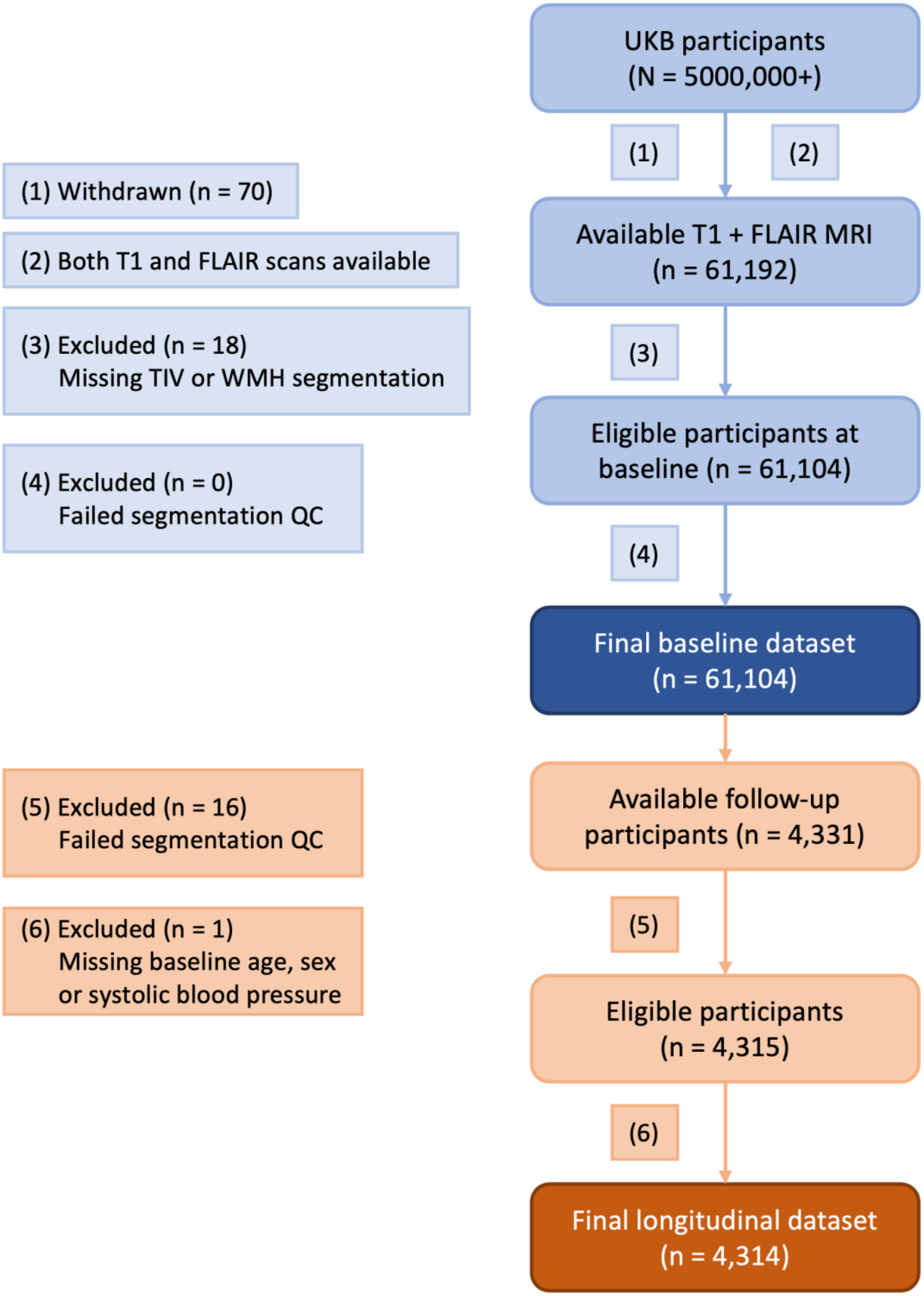

eFigure 5 Flowchart for OASIS-3 participants

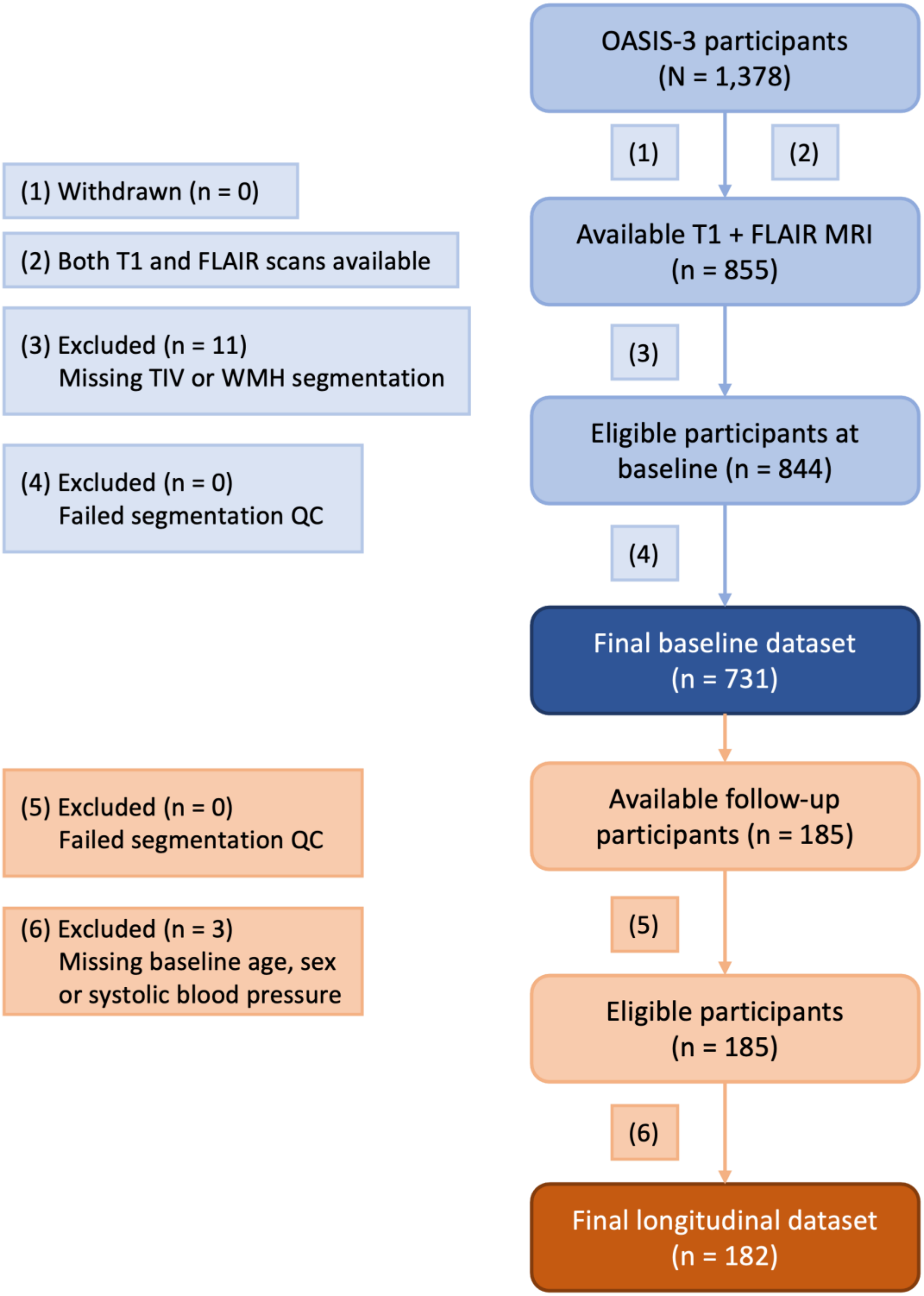

e2.2 Baseline characteristics by cohort

eTable 6 Baseline characteristics across cohorts.

	CATEGORY	UNIT	ADNI-3 (N=1,007)	INSIGHT46 (N=462)	SABRE (N=765)	UKB (N=61,104)	OASIS-3 (N=844)
Age[a]		N[b]	984	462	476	61,104	731
		years	72.8 [67.4, 78.6]	70.7 [70.1, 71.2]	72.6 [69.2, 76.9]	65.9 [59.7, 71.4]	71.3 [65.9, 76.2]
Sex[c]		N	987	462	736	61,104	729
	Female	n (%)	520 (52.7%)	226 (48.9%)	329 (44.7%)	32,326 (52.9%)	488 (66.9%)
	Male	n (%)	467 (47.3%)	236 (51.1%)	407 (55.3%)	28,778 (47.1%)	241 (33.1%)
Ethnicity		N	978	–	736	60,932	736
	Asian	n (%)	30 (3.1%)	–	253 (34.4%)	854 (1.4%)	< 10[d]
	Black	n (%)	106 (10.8%)	–	171 (23.2%)	421 (0.7%)	106 (14.4%)
	Mixed	n (%)	21 (2.1%)	–	–	321 (0.5%)	< 10[d]
	White	n (%)	819 (83.7%)	–	308 (41.8%)	58,988 (96.8%)	622 (84.5%)
	Other	n (%)	< 10[d]	–	< 10[d]	348 (0.6%)	–
Total baseline WMH volume		N	1,007	462	765	61,104	844
		mL	3.2 [1.4, 8.3]	3.7 [1.9, 7.8]	2.9 [1.6, 8.0]	1.8 [1.1, 3.8]	2.5 [1.3, 5.8]
APOE4		N	796	462	736	57,298	732
	None	n (%)	495 (62.2%)	323 (69.9%)	617 (83.8%)	41,522 (72.5%)	437 (59.7%)
	1 allele	n (%)	250 (31.4%)	127 (27.5%)	106 (14.4%)	14,540 (25.4%)	254 (34.7%)
	2 alleles	n (%)	51 (6.4%)	12 (2.6%)	13 (1.8%)	1,236 (2.2%)	41 (5.6%)
Smoking		N	–	455	635	60,439	566
	Current	n (%)	–	16 (3.5%)	22 (3.5%)	2,023 (3.3%)	36 (6.4%)
	Ever	n (%)	–	281 (61.8%)	219 (34.5%)	20,380 (33.7%)	226 (39.9%)
	Never	n (%)	–	158 (34.7%)	394 (62.0%)	38,036 (62.9%)	304 (53.7%)
Hypertension		N	987	22	736	61,104	451
	No	n (%)	555 (56.2%)	< 10[d]	292 (39.7%)	42,901 (70.2%)	239 (53.0%)
	Yes	n (%)	432 (43.8%)	21 (95.5%)	444 (60.3%)	18,203 (29.8%)	212 (47.0%)
Anti-hypertensive medication		N	–	462	736	60,360	736
	No	n (%)	–	275 (59.5%)	310 (42.1%)	45,331 (75.1%)	483 (65.6%)
	Yes	n (%)	–	187 (40.5%)	426 (57.9%)	15,029 (24.9%)	253 (34.4%)
Systolic blood pressure		N	973	461	735	61,074	728
		mmHg	133.0 [121.0, 145.0]	137.5 [126.5, 148.0]	140.0 [129.0, 152.5]	135.0 [123.0, 148.0]	130.0 [118.0, 140.0]
Diastolic blood pressure		N	973	461	735	61,074	703
		mmHg	74.0 [68.0, 80.0]	73.0 [66.5, 80.5]	79.0 [72.0, 86.0]	81.0 [74.0, 88.0]	76.0 [70.0, 81.5]
Type 2 diabetes		N	–	462	736	61,104	452
	No	n (%)	–	411 (89.0%)	641 (87.1%)	58,558 (95.8%)	403 (89.2%)
	Yes	n (%)	–	51 (11.0%)	95 (12.9%)	2,546 (4.2%)	49 (10.8%)
Diabetic medication		N	–	462	736	60,360	736
	No	n (%)	–	428 (92.6%)	604 (82.1%)	59,911 (99.3%)	674 (91.6%)
	Yes	n (%)	–	34 (7.4%)	132 (17.9%)	449 (0.7%)	62 (8.4%)
HbA1c		N	–	462	719	56,727	–
		mmol/mol	–	38.0 [36.0, 41.0]	38.8 [34.7, 43.8]	34.5 [32.2, 37.0]	–
Glucose		N	659	458	723	35,383	–
		mmol/L	5.3 [5.0, 6.0]	4.9 [4.4, 5.4]	4.4 [4.0, 4.9]	3.5 [3.1, 4.0]	–
Lipid-lowering medication		N	–	462	736	60,360	736
	No	n (%)	–	294 (63.6%)	359 (48.8%)	45,108 (74.7%)	394 (53.5%)
	Yes	n (%)	–	168 (36.4%)	377 (51.2%)	15,252 (25.3%)	342 (46.5%)
LDL		N	–	462	721	35,445	–
		mmol/L	–	3.0 [2.3, 3.8]	2.4 [1.8, 3.1]	1.7 [1.5, 2.0]	–
HDL		N	–	462	727	35,445	–
		mmol/L	–	1.6 [1.3, 2.0]	1.5 [1.3, 1.9]	1.3 [1.1, 1.5]	–
Triglycerides		N	659	415	727	35,445	–
		mmol/L	1.3 [1.0, 1.8]	1.2 [0.9, 1.6]	1.2 [0.9, 1.6]	1.1 [0.8, 1.6]	–
WBCC		N	–	462	726	58,269	–
		10^9/L	–	6.5 [5.5, 7.8]	6.3 [5.3, 7.7]	6.4 [5.4, 7.5]	–
Creatinine		N	659	458	727	34,565	–
		μmol/L	80.4 [69.0, 93.7]	73.0 [64.0, 84.0]	78.0 [67.0, 91.0]	65.7 [57.7, 74.6]	–

a. Continuous variables reported as median [IQR].
b. For each variable, the preceding N row is the number of non-missing observations available in that cohort.
c. Categorical variables are reported as counts (%).
d. Categories with small counts were suppressed as < 10 to reduce disclosure risk.

e2.3 Comparison of internal dataset and OASIS-3

eTable 7 Median and IQR of total WMH volume (mL) of each cluster in the internal dataset and OASIS-3.

DATASET	CLUSTER 1	CLUSTER 2	CLUSTER 3	CLUSTER 4	CLUSTER 5
Internal	0.9 [0.7, 1.3]mL	1.5 [1.0, 2.1]mL	1.9 [1.2, 3.0]mL	4.4 [2.5, 7.8]mL	6.4 [3.5, 11.9]mL
OASIS-3	0.8 [0.5, 1.2]mL	1.5 [1.1, 2.4]mL	1.7 [1.1, 3.0]mL	5.2 [2.5, 10.5]mL	6.0 [3.2, 11.9]mL

e2.4 Identification of location patterns

The stability profiles of all clustering methods are shown in eFigure 6 below for k between 2 and 7. Using a stability threshold of 0.90, subKMeans achieved the highest stability of 0.946 when $k = 5$ (0.944 ± 0.001 in 5-fold cross-validation), supporting the selection of $k = 5$ for subKMeans as the optimal number of clusters for the dataset. Representative participants from each location pattern are also shown.

eFigure 6 Stability profiles of different clustering methods, averaged over 5-fold cross-validations.

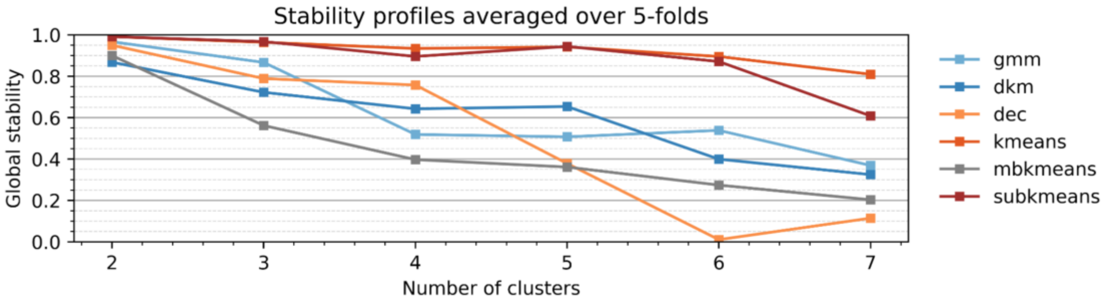

eFigure 7 Proportions of (a) sex and (b) age groups (below 65, and 65 and above) in each cluster in the internal dataset.

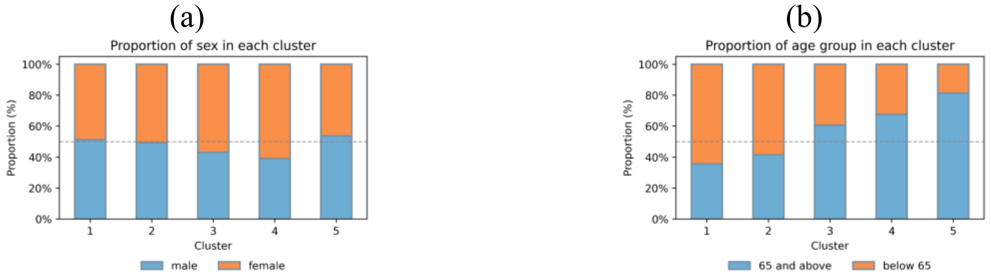

e2.5 Within-participant transitions between WMH location patterns

eTable 8 Among individuals with 18-30 months follow-up interval, within-participant transitions in WMH location patterns between baseline and follow-up.

MOVEMENT TYPE	NUMBER OF PARTICIPANTS	PERCENT (%)
Stayed in same cluster	2,697	71.5
Moved within low-burden clusters 1-3	706	18.7
Moved within high-burden clusters 4-5	59	1.6
Moved 1/2/3 → 4/5	235	6.2
Moved 4/5 → 1/2/3	75	2.0
Total	3,772	100.0

e2.6 Cross-sectional association between location patterns and risk factors

Internal dataset

*eTable 9 Cross-sectional associations between risk factors and proximity to centroids of 5 clusters the internal dataset, estimated using multivariable linear regression and adjusted by age, sex and total WMH volume. β is the standardised effect size, L and U are the lower and upper limits of the 95% CI. P-values were BH-corrected. ** indicates p < 0.001. Abbreviations: WMHV=total WMH volume, SBP/DBP=systolic/diastolic blood pressure, DM2=type 2 diabetes, MedChol=lipid-lowering medication, MedBP=anti-hypertensive medication, MedDM=diabetic medication, HDL=high-density lipoprotein cholesterol, LDL=low-density lipoprotein cholesterol, WBCC=white blood cell count. Categorical variables and reference categories: sex (male), smoking (never), ethnicity (white), number of APOE4 alleles (none), and diagnoses of medication conditions or use of medications (no).*

RISK FACTOR	CATEGORY	CLUSTER 1				CLUSTER 2				CLUSTER 3				CLUSTER 4				CLUSTER 5			
		β	L	U	P-VALUE	β	L	U	P-VALUE	β	L	U	P-VALUE	β	L	U	P-VALUE	β	L	U	P-VALUE
WMHV		-2.404	-2.453	-2.356	**	-1.816	-1.875	-1.758	**	-1.461	-1.525	-1.397	**	2.792	2.731	2.854	**	4.243	4.184	4.301	**
Age		-0.446	-0.462	-0.431	**	-0.320	-0.338	-0.302	**	0.203	0.183	0.223	**	0.393	0.374	0.413	**	0.545	0.527	0.564	**
Sex	Female	-0.012	-0.016	-0.007	**	-0.017	-0.022	-0.011	**	0.040	0.035	0.046	**	0.054	0.049	0.060	**	-0.015	-0.021	-0.010	**
Ethnicity	Other	0.001	-0.028	0.029	0.969	-0.037	-0.071	-0.002	0.058	-0.004	-0.042	0.034	0.876	-0.015	-0.051	0.021	0.501	-0.015	-0.049	0.019	0.485
Ethnicity	Mixed	-0.021	-0.050	0.008	0.221	-0.026	-0.060	0.009	0.221	-0.042	-0.080	-0.003	0.053	-0.024	-0.060	0.013	0.283	-0.002	-0.036	0.033	0.940
Ethnicity	Asian	-0.028	-0.044	-0.011	0.002	-0.054	-0.074	-0.034	**	-0.061	-0.083	-0.039	**	-0.039	-0.060	-0.019	**	-0.021	-0.041	-0.001	0.060
Ethnicity	Black	-0.112	-0.134	-0.090	**	-0.073	-0.099	-0.047	**	-0.107	-0.136	-0.078	**	-0.013	-0.041	0.014	0.442	0.048	0.022	0.074	0.001
APOE4	One	-0.003	-0.008	0.002	0.295	0.003	-0.003	0.009	0.422	0.001	-0.006	0.008	0.814	-0.001	-0.007	0.006	0.902	0.001	-0.005	0.007	0.711
APOE4	Two	-0.040	-0.055	-0.025	**	0.013	-0.005	0.031	0.221	-0.048	-0.067	-0.028	**	-0.040	-0.059	-0.021	**	-0.009	-0.026	0.009	0.440
Smoking	Current	-0.021	-0.033	-0.009	0.002	-0.011	-0.026	0.003	0.195	-0.004	-0.020	0.012	0.696	0.004	-0.011	0.019	0.678	0.021	0.006	0.035	0.009
Smoking	Ever	-0.009	-0.014	-0.004	**	-0.009	-0.015	-0.004	0.002	0.004	-0.002	0.010	0.254	0.002	-0.004	0.008	0.589	0.004	-0.002	0.009	0.221
Hypertension	Yes	-0.033	-0.037	-0.028	**	-0.040	-0.045	-0.034	**	0.013	0.007	0.020	**	0.055	0.049	0.061	**	0.031	0.025	0.037	**
MedBP	Yes	-0.031	-0.036	-0.026	**	-0.041	-0.047	-0.035	**	0.013	0.007	0.020	**	0.062	0.055	0.068	**	0.033	0.027	0.039	**
DBP		-0.091	-0.111	-0.071	**	-0.151	-0.175	-0.126	**	0.121	0.094	0.148	**	0.245	0.219	0.271	**	0.164	0.139	0.188	**
SBP		-0.101	-0.123	-0.079	**	-0.117	-0.143	-0.091	**	0.091	0.062	0.120	**	0.241	0.213	0.268	**	0.166	0.140	0.193	**
DM2	Yes	-0.035	-0.045	-0.024	**	-0.039	-0.052	-0.027	**	0.020	0.006	0.034	0.008	0.015	0.002	0.029	0.042	0.003	-0.010	0.015	0.719
MedDM	Yes	-0.065	-0.088	-0.043	**	-0.041	-0.068	-0.014	0.005	0.006	-0.024	0.035	0.764	-0.011	-0.039	0.018	0.538	0.015	-0.012	0.042	0.367
HbA1c		-0.139	-0.233	-0.045	0.007	-0.030	-0.144	0.084	0.678	-0.038	-0.163	0.086	0.628	0.007	-0.111	0.126	0.926	0.047	-0.065	0.159	0.496
Glucose		-0.259	-0.322	-0.196	**	-0.065	-0.140	0.011	0.144	-0.063	-0.146	0.020	0.205	-0.089	-0.168	-0.010	0.046	0.076	0.001	0.151	0.077
MedChol	Yes	-0.019	-0.024	-0.014	**	-0.032	-0.038	-0.025	**	0.011	0.004	0.018	0.002	0.031	0.025	0.038	**	0.009	0.003	0.015	0.009
LDL		-0.079	-0.177	0.019	0.178	0.062	-0.057	0.181	0.395	-0.041	-0.172	0.089	0.618	-0.009	-0.133	0.115	0.918	0.056	-0.062	0.173	0.442
HDL		-0.079	-0.190	0.031	0.221	0.096	-0.037	0.229	0.221	-0.061	-0.207	0.084	0.496	-0.036	-0.175	0.102	0.678	0.061	-0.070	0.192	0.449
Triglycerides		-0.114	-0.161	-0.067	**	-0.276	-0.333	-0.219	**	0.081	0.018	0.143	0.020	0.157	0.098	0.217	**	-0.082	-0.139	-0.025	0.008
Creatinine		-0.246	-0.321	-0.171	**	-0.314	-0.404	-0.223	**	-0.238	-0.338	-0.138	**	-0.186	-0.281	-0.090	**	-0.235	-0.325	-0.144	**
WBCC		-0.108	-0.203	-0.013	0.044	0.002	-0.113	0.118	0.969	-0.079	-0.206	0.047	0.296	0.016	-0.105	0.136	0.848	0.062	-0.053	0.176	0.382

OASIS-3

eTable 10 Cross-sectional associations between risk factors and proximity to centroids of 5 clusters the OASIS-3, estimated using multivariable linear regression and adjusted by age, sex and total WMH volume. β is the standardised effect size, L and U are the lower and upper limits of the 95% CI. P-values were BH-corrected. Abbreviations: WMHV=total WMH volume, SBP/DBP=systolic/diastolic blood pressure, DM2=type 2 diabetes, MedChol=lipid-lowering medication, MedBP=anti-hypertensive medication, MedDM=diabetic medication, HDL=high-density lipoprotein cholesterol, LDL=low-density lipoprotein cholesterol. Categorical variables and reference categories: sex (male), smoking (never), ethnicity (white), number of APOE4 alleles (none), and diagnoses of medication conditions or use of medications (no).

		CLUSTER 1				CLUSTER 2				CLUSTER 3				CLUSTER 4				CLUSTER 5			
**RISK FACTOR**	**CATEGORY**	**β**	**L**	**U**	**P-VALUE**	**β**	**L**	**U**	**P-VALUE**	**β**	**L**	**U**	**P-VALUE**	**β**	**L**	**U**	**P-VALUE**	**β**	**L**	**U**	**P-VALUE**
**WMHV**		-0.681	-0.835	-0.528	0.000	-0.992	-1.193	-0.791	0.000	-0.873	-1.087	-0.658	0.000	0.538	0.362	0.714	0.000	0.831	0.587	1.074	0.000
**Age**		-0.285	-0.372	-0.197	0.000	-0.161	-0.276	-0.046	0.039	0.000	-0.123	0.123	1.000	0.226	0.125	0.326	0.000	0.652	0.513	0.791	0.000
**Sex**	**Female**	-0.016	-0.045	0.013	0.560	0.037	-0.001	0.075	0.214	-0.042	-0.083	-0.002	0.183	-0.025	-0.059	0.008	0.377	0.078	0.032	0.124	0.008
**Ethnicity**	**Other**										Insufficient participants										
**Ethnicity**	**Mixed**	-0.125	-0.336	0.085	0.521	0.003	-0.272	0.278	1.000	-0.148	-0.444	0.147	0.588	-0.163	-0.407	0.081	0.451	-0.292	-0.628	0.044	0.285
**Ethnicity**	**Asian**	-0.109	-0.273	0.054	0.451	-0.200	-0.413	0.013	0.238	-0.014	-0.243	0.216	1.000	-0.022	-0.211	0.168	0.950	-0.115	-0.376	0.146	0.622
**Ethnicity**	**Black**	-0.077	-0.116	-0.038	0.001	-0.113	-0.164	-0.063	0.000	-0.086	-0.140	-0.032	0.014	-0.028	-0.073	0.016	0.469	-0.056	-0.117	0.006	0.258
**APOE4**	**One**	-0.028	-0.057	0.001	0.214	-0.002	-0.041	0.036	1.000	-0.015	-0.055	0.026	0.727	-0.003	-0.037	0.030	0.969	0.023	-0.023	0.070	0.588
**APOE4**	**Two**	-0.086	-0.147	-0.026	0.036	-0.022	-0.101	0.058	0.813	-0.104	-0.188	-0.019	0.085	-0.017	-0.087	0.052	0.839	0.101	0.005	0.197	0.183
**Smoking**	**Current**	-0.012	-0.078	0.055	0.866	-0.042	-0.129	0.044	0.588	0.068	-0.022	0.159	0.377	0.077	0.000	0.154	0.199	0.038	-0.065	0.142	0.727
**Smoking**	**Ever**	0.016	-0.018	0.049	0.603	-0.008	-0.051	0.036	0.866	0.060	0.014	0.106	0.057	0.030	-0.008	0.069	0.375	0.044	-0.008	0.096	0.303
**Hypertension**	**Yes**	-0.019	-0.053	0.016	0.562	-0.051	-0.097	-0.005	0.148	-0.007	-0.057	0.042	0.894	0.024	-0.018	0.066	0.536	0.012	-0.047	0.071	0.866
**MedBP**	**Yes**	-0.009	-0.038	0.020	0.774	-0.012	-0.050	0.026	0.774	-0.007	-0.048	0.033	0.866	0.000	-0.033	0.034	1.000	0.025	-0.021	0.071	0.560
**DBP**		-0.048	-0.130	0.034	0.525	0.030	-0.076	0.137	0.809	-0.023	-0.136	0.090	0.866	0.045	-0.048	0.139	0.590	0.091	-0.039	0.221	0.445
**SBP**		-0.080	-0.185	0.025	0.377	0.005	-0.133	0.142	1.000	-0.035	-0.181	0.112	0.842	0.027	-0.094	0.147	0.854	0.172	0.006	0.338	0.183
**DM2**	**Yes**	0.010	-0.044	0.063	0.866	-0.019	-0.091	0.052	0.813	0.036	-0.040	0.112	0.597	0.044	-0.020	0.109	0.451	0.030	-0.062	0.122	0.774
**MedDM**	**Yes**	0.012	-0.038	0.061	0.842	-0.023	-0.088	0.041	0.727	0.047	-0.022	0.115	0.451	0.051	-0.005	0.107	0.258	0.035	-0.043	0.112	0.622
**MedChol**	**Yes**	-0.014	-0.041	0.014	0.588	-0.011	-0.048	0.025	0.774	0.025	-0.014	0.064	0.467	0.042	0.010	0.073	0.057	0.028	-0.016	0.072	0.469

e2.7 Baseline characteristics of the longitudinal dataset

eTable 11 Baseline characteristics comparing non-progression and progression groups in the Internal and OASIS-3 datasets.

	CATEGORY	UNIT	INTERNAL			OASIS-3		
			Non-progressor (n=3,779)	Progressor (n=1,495)	P-value[c]	Non-progressor (n=94)	Progressor (n=88)	P-value[c]
Age[a]		years	62.2 [55.8, 69.2]	68.8 [62.1, 73.3]	< 0.001	65.5 [59.3, 70.6]	71.7 [68.1, 76.5]	< 0.001
Sex[b]					0.739			0.629
	Female	n	1,997 (52.8%)	779 (52.1%)		60 (63.8%)	62 (70.5%)	
	Male	n	1,782 (47.2%)	716 (47.9%)		34 (36.2%)	26 (29.5%)	
Ethnicity					0.602			0.798
	Asian	n	44 (1.3%)	17 (1.2%)		< 10[d]	–	
	Black	n	36 (1.0%)	29 (2.0%)		10 (10.6%)	11 (12.5%)	
	Mixed	n	20 (0.6%)	11 (0.7%)		–	–	
	White	n	3,324 (96.7%)	1,412 (95.9%)		83 (88.3%)	77 (87.5%)	
	Other	n	14 (0.4%)	< 10[d]		–	–	
Total baseline WMH volume		mL	1.4 [1.0, 2.4]	3.8 [1.8, 7.2]	< 0.001	1.5 [0.9, 2.4]	4.1 [2.5, 7.5]	0.064
dist1		–	0.73 [0.63, 0.85]	0.90 [0.76, 1.03]	< 0.001	0.90 [0.79, 1.01]	1.01 [0.91, 1.13]	0.049
dist2		–	0.71 [0.60, 0.84]	0.79 [0.67, 0.93]	< 0.001	0.78 [0.67, 0.89]	0.89 [0.77, 0.99]	0.166
dist3		–	0.74 [0.63, 0.86]	0.75 [0.62, 0.90]	0.858	0.80 [0.69, 0.93]	0.83 [0.72, 0.96]	0.820
dist4		–	0.89 [0.75, 1.01]	0.72 [0.60, 0.86]	< 0.001	0.91 [0.79, 1.01]	0.81 [0.74, 0.90]	0.080
dist5		–	0.94 [0.79, 1.07]	0.72 [0.58, 0.88]	< 0.001	0.93 [0.76, 1.02]	0.72 [0.59, 0.82]	< 0.001
APOE4					0.005			0.347
	None	n	2,570 (72.4%)	949 (69.2%)		65 (69.9%)	58 (65.9%)	
	1 allele	n	911 (25.6%)	369 (26.9%)		27 (29.0%)	24 (27.3%)	
	2 alleles	n	71 (2.0%)	53 (3.9%)		< 10[d]	< 10[d]	
Smoking					0.858			0.925
	Current	n	121 (3.5%)	36 (3.2%)		< 10[d]	< 10[d]	
	Ever	n	1,171 (33.4%)	419 (37.2%)		26 (31.7%)	28 (41.8%)	
	Never	n	2,212 (63.1%)	671 (59.6%)		49 (59.8%)	33 (49.3%)	
Hypertension					0.004			0.518
	No	n	2,620 (75.7%)	938 (63.4%)		14 (77.8%)	16 (51.6%)	
	Yes	n	841 (24.3%)	541 (36.6%)		< 10[d]	15 (48.4%)	
Anti-hypertensive medication					0.156			0.489
	No	n	2,833 (80.8%)	828 (73.3%)		71 (75.5%)	52 (59.1%)	
	Yes	n	672 (19.2%)	302 (26.7%)		23 (24.5%)	36 (40.9%)	
Systolic blood pressure		mmHg	132.0 [121.0, 144.0]	138.0 [126.0, 150.0]	< 0.001	124.0 [117.2, 135.8]	130.0 [118.0, 139.2]	0.518
Diastolic blood pressure		mmHg	79.0 [72.0, 86.0]	80.0 [73.0, 88.0]	< 0.001	76.0 [70.0, 80.0]	76.0 [70.0, 81.8]	0.347
Type 2 diabetes					0.189			0.418
	No	n	3,417 (96.9%)	1,083 (95.2%)		14 (77.8%)	29 (93.5%)	
	Yes	n	111 (3.1%)	55 (4.8%)		< 10[d]	< 10[d]	
Diabetic medication					0.189			0.418
	No	n	3,464 (98.8%)	1,121 (99.2%)		85 (90.4%)	81 (92.0%)	
	Yes	n	41 (1.2%)	< 10[d]		< 10[d]	< 10[d]	
HbA1c		mmol/mol	34.300 [32.000, 36.900]	34.800 [32.600, 37.400]	< 0.001	–	–	–
Glucose		mmol/L	3.644 [3.171, 4.300]	3.819 [3.278, 4.799]	0.215	–	–	–
Lipid-lowering medication					0.006			0.820
	No	n	2,884 (82.3%)	809 (71.6%)		63 (67.0%)	50 (56.8%)	
	Yes	n	621 (17.7%)	321 (28.4%)		31 (33.0%)	38 (43.2%)	
HDL		mmol/L	1.350 [1.128, 1.600]	1.295 [1.116, 1.552]	< 0.001	–	–	–
LDL		mmol/L	1.797 [1.501, 2.160]	1.739 [1.482, 2.050]	< 0.001	–	–	–
Triglycerides		mmol/L	1.135 [0.845, 1.586]	1.227 [0.854, 1.631]	0.858	–	–	–
White blood cell count		10^9/L	6.330 [5.400, 7.500]	6.330 [5.400, 7.470]	< 0.001	–	–	–
Creatinine		μmol/L	67.596 [58.732, 77.555]	68.776 [59.481, 79.663]	0.156	–	–	–

a. For continuous variables, Median [IQR] are provided and between-group differences were compared using Mann-Whitney U test after removing the effects of age and sex.
b. For categorical variables, counts of participants (%) are provided, and between-group differences were compared using Chi-squared test after removing the effects of age and sex.
c. P-values were BH-corrected in internal dataset and OASIS-3 respectively.
d. Categories with small counts suppressed as < 10.

e2.8 Longitudinal prediction

eTable 12 Model performance for WMH progression prediction, reported as balanced accuracy (mean and 95% CI) across train, test, and OASIS-3 datasets. Abbreviations: clusters = spatial patterns of WMH, distance = Euclidean distance to five WMH cluster centroids, RF = age, sex, and systolic blood pressure, WMHV = total baseline WMH volume, RWMHV = 36 regional baseline WMH volume, PCV = principal components of RWMHV.

MODEL	TRAIN (95% CI)			TEST (95% CI)			OASIS-3 (95% CI)		
	MEAN	LOWER	UPPER	MEAN	LOWER	UPPER	MEAN	LOWER	UPPER
RF	0.632	0.617	0.646	0.657	0.626	0.686	0.687	0.622	0.751
WMHV	0.707	0.680	0.734	0.693	0.664	0.723	0.748	0.688	0.804
PCV	0.724	0.704	0.744	0.693	0.662	0.722	0.777	0.718	0.831
clusters	0.696	0.680	0.712	0.649	0.619	0.677	0.689	0.626	0.750
distance	0.729	0.712	0.747	0.712	0.681	0.742	0.711	0.653	0.769
WMHV + RF	0.729	0.706	0.751	0.706	0.676	0.735	0.778	0.717	0.840
PCV + RF	0.737	0.713	0.762	0.702	0.670	0.730	0.785	0.727	0.846
clusters + RF	0.716	0.690	0.742	0.700	0.669	0.730	0.697	0.635	0.759
distance + RF	0.735	0.699	0.772	0.709	0.678	0.738	0.706	0.647	0.769
WMHV + PCV	0.733	0.708	0.758	0.695	0.664	0.724	0.795	0.736	0.848
WMHV + clusters	0.720	0.703	0.738	0.692	0.662	0.722	0.752	0.689	0.813
WMHV + distance	0.736	0.718	0.754	0.712	0.680	0.742	0.727	0.664	0.787
WMHV + PCV + RF	0.739	0.721	0.757	0.710	0.679	0.740	0.791	0.735	0.849
WMHV + clusters + RF	0.736	0.711	0.760	0.701	0.669	0.730	0.752	0.694	0.815
WMHV + distance + RF	0.743	0.724	0.763	0.707	0.677	0.736	0.732	0.669	0.790
RWMHV	0.745	0.730	0.760	0.733	0.703	0.762	0.741	0.676	0.801
RWMHV + RF	0.749	0.742	0.756	0.730	0.701	0.757	0.741	0.678	0.801
RWMHV + PCV	0.745	0.734	0.756	0.728	0.699	0.756	0.757	0.695	0.814
RWMHV + clusters	0.753	0.734	0.772	0.733	0.702	0.760	0.741	0.677	0.802
RWMHV + distance	0.742	0.728	0.757	0.734	0.704	0.761	0.741	0.678	0.798
RWMHV + PCV + RF	0.748	0.739	0.757	0.737	0.706	0.764	0.764	0.705	0.821
RWMHV + clusters + RF	0.745	0.717	0.773	0.725	0.695	0.752	0.725	0.659	0.791
RWMHV + distance + RF	0.748	0.736	0.759	0.736	0.707	0.762	0.741	0.674	0.800

Internal dataset

*eTable 13 BH-corrected p-values from pairwise McNemar's tests between models for WMH progression in internal test set. bold indicates p-values < 0.05, ** indicates p-values < 0.001. Abbreviations: clusters=location patterns of WMH, distance=Euclidean distance to five cluster centroids, RF=age, sex and systolic blood pressure, WMHV=total baseline WMH volume, RWMHV=36 regional baseline WMH volume, PCV=principal components of RWMHV.*

	RF	WMHV	PCV	CLUSTERS	DISTANCE	WMHV + RF	PCV + RF	CLUSTERS + RF	DISTANCE + RF	WMHV + PCV	WMHV + CLUSTERS	WMHV + DISTANCE	WMHV + PCV + RF	WMHV + CLUSTERS + RF	WMHV + DISTANCE + RF	RWMHV	RWMHV + RF	RWMHV + PCV	RWMHV + CLUSTERS	RWMHV + DISTANCE	RWMHV + PCV + RF	RWMHV + CLUSTERS + RF	RWMHV + DISTANCE + RF
**RF**		**0.015**	**0.030**	**0.027**	**0.025**	**	**0.005**	0.148	**0.022**	**0.026**	0.076	**0.004**	**0.002**	**0.003**	**0.006**	**	**	**	**	**	**	**	**
**WMHV**	**0.016**		0.664	**	0.816	0.136	0.973	0.168	0.763	0.771	0.334	0.763	0.763	1.000	1.000	**0.023**	**0.046**	0.089	0.060	0.100	**0.046**	0.124	0.089
**PCV**	**0.030**	0.664		**	1.000	**0.042**	0.454	0.292	1.000	0.938	0.694	0.369	0.252	0.664	0.669	**0.002**	**0.006**	**0.011**	**0.008**	**0.017**	**0.004**	**0.022**	**0.017**
**CLUSTERS**	**0.027**	**	**		**	**	**	**	**	**	**	**	**	**	**	**	**	**	**	**	**	**	**
**DISTANCE**	**0.025**	0.816	1.000	**		0.160	0.712	0.242	0.973	1.000	0.709	0.296	0.536	0.763	0.709	**0.006**	**0.009**	**0.026**	**0.011**	**0.014**	**0.009**	**0.027**	**0.017**
**WMHV + RF**	**	0.136	**0.042**	**	0.160		0.116	**0.006**	0.110	0.076	**0.027**	0.559	0.297	0.093	0.176	0.263	0.370	0.669	0.490	0.646	0.388	0.704	0.587
**PCV + RF**	**0.005**	0.973	0.454	**	0.712	0.116		0.057	0.604	0.631	0.296	0.816	0.669	1.000	1.000	**0.021**	**0.026**	0.071	**0.045**	0.082	**0.018**	0.081	0.068
**CLUSTERS + RF**	0.148	0.168	0.292	**	0.242	**0.006**	0.057		0.223	0.247	0.560	**0.040**	**0.031**	**0.042**	0.055	**	**	**	**	**	**	**	**
**DISTANCE + RF**	**0.022**	0.763	1.000	**	0.973	0.110	0.604	0.223		0.958	0.808	0.308	0.405	0.656	0.567	**0.004**	**0.004**	**0.019**	**0.007**	**0.014**	**0.004**	**0.014**	**0.008**
**WMHV + PCV**	**0.026**	0.771	0.938	**	1.000	0.076	0.631	0.247	0.958		0.587	0.481	0.340	0.763	0.768	**0.005**	**0.011**	**0.019**	**0.016**	**0.030**	**0.007**	**0.037**	**0.030**
**WMHV + CLUSTERS**	0.076	0.334	0.694	**	0.709	**0.027**	0.296	0.560	0.808	0.587		0.121	0.176	0.323	0.353	**	**0.004**	**0.006**	**0.004**	**0.008**	**0.003**	**0.013**	**0.008**
**WMHV + DISTANCE**	**0.004**	0.763	0.369	**	0.296	0.559	0.816	**0.040**	0.308	0.481	0.121		1.000	0.775	0.709	**0.038**	0.075	0.155	0.082	0.118	0.075	0.191	0.124
**WMHV + PCV + RF**	**0.002**	0.763	0.252	**	0.536	0.297	0.669	**0.031**	0.405	0.340	0.176	1.000		0.771	0.784	**0.038**	**0.046**	0.135	0.081	0.143	**0.030**	0.148	0.117
**WMHV + CLUSTERS + RF**	**0.004**	1.000	0.664	**	0.763	0.093	1.000	**0.042**	0.656	0.763	0.323	0.775	0.771		0.973	**0.021**	**0.027**	0.081	**0.044**	0.082	**0.024**	0.082	0.064
**WMHV + DISTANCE + RF**	**0.006**	1.000	0.669	**	0.709	0.176	1.000	0.055	0.567	0.768	0.353	0.709	0.784	0.973		**0.017**	**0.022**	0.067	**0.037**	0.055	**0.021**	0.061	**0.040**
**RWMHV**	**	**0.023**	**0.002**	**	**0.006**	0.263	**0.021**	**	**0.004**	**0.005**	**	**0.038**	**0.038**	**0.021**	**0.017**		0.810	0.413	0.676	0.454	0.776	0.370	0.588
**RWMHV + RF**	**	**0.046**	**0.006**	**	**0.009**	0.370	**0.026**	**	**0.004**	**0.011**	**0.004**	0.075	**0.046**	**0.027**	**0.022**	0.810		0.692	0.970	0.763	1.000	0.516	0.808
**RWMHV + PCV**	**	0.089	**0.011**	**	**0.026**	0.669	0.071	**	**0.019**	**0.019**	**0.006**	0.155	0.135	0.081	0.067	0.413	0.692		0.809	1.000	0.737	1.000	0.973
**RWMHV + CLUSTERS**	**	0.060	**0.008**	**	**0.011**	0.490	**0.045**	**	**0.007**	**0.016**	**0.004**	0.082	0.081	**0.044**	**0.037**	0.676	0.970	0.809		0.881	1.000	0.733	0.970
**RWMHV + DISTANCE**	**	0.100	**0.017**	**	**0.014**	0.646	0.082	**	**0.014**	**0.030**	**0.008**	0.118	0.143	0.082	0.055	0.454	0.763	1.000	0.881		0.834	0.970	1.000
**RWMHV + PCV + RF**	**	**0.046**	**0.004**	**	**0.009**	0.388	**0.018**	**	**0.004**	**0.007**	**0.003**	0.075	**0.030**	**0.024**	**0.021**	0.776	1.000	0.737	1.000	0.834		0.689	0.893
**RWMHV + CLUSTERS + RF**	**	0.124	**0.022**	**	**0.027**	0.704	0.081	**	**0.014**	**0.037**	**0.013**	0.191	0.148	0.082	0.061	0.370	0.516	1.000	0.733	0.970	0.689		0.881
**RWMHV + DISTANCE + RF**	**	0.089	**0.017**	**	**0.017**	0.587	0.068	**	**0.008**	**0.030**	**0.008**	0.124	0.117	0.064	**0.040**	0.588	0.808	0.973	0.970	1.000	0.893	0.881	

OASIS-3

*eTable 14 BH-corrected p-values from pairwise McNemar's tests between models for WMH progression in OASIS-3. bold indicates p-values < 0.05, ** indicates p-values < 0.001. Abbreviations: clusters=location patterns of WMH, distance=Euclidean distance to five cluster centroids, RF=age, sex and systolic blood pressure, WMHV=total baseline WMH volume, RWMHV=36 regional baseline WMH volume, PCV=principal components of RWMHV.*

	RF	WMHV	PCV	CLUSTERS	DISTANCE	WMHV + RF	PCV + RF	CLUSTERS + RF	DISTANCE + RF	WMHV + PCV	WMHV + CLUSTERS	WMHV + DISTANCE	WMHV + PCV + RF	WMHV + CLUSTERS + RF	WMHV + DISTANCE + RF	RWMHV	RWMHV + RF	RWMHV + PCV	RWMHV + CLUSTERS	RWMHV + DISTANCE	RWMHV + PCV + RF	RWMHV + CLUSTERS + RF	RWMHV + DISTANCE + RF
**RF**		0.556	0.317	0.945	0.890	0.227	0.213	0.922	0.922	0.213	0.523	0.688	0.213	0.393	0.620	0.588	0.588	0.469	0.588	0.588	0.342	0.661	0.584
**WMHV**	0.556		0.636	0.504	0.635	0.677	0.622	0.584	0.616	0.435	0.922	0.801	0.588	0.922	0.868	0.922	0.922	1.000	0.922	0.922	0.922	0.788	0.922
**PCV**	0.317	0.636		0.227	0.355	0.922	1.000	0.307	0.325	0.818	0.724	0.471	0.922	0.742	0.504	0.620	0.620	0.792	0.620	0.620	0.899	0.453	0.610
**CLUSTERS**	0.945	0.504	0.227		0.868	0.227	0.213	0.922	0.922	0.213	0.317	0.636	0.213	0.445	0.616	0.588	0.588	0.423	0.584	0.588	0.342	0.644	0.556
**DISTANCE**	0.890	0.635	0.355	0.868		0.342	0.227	0.922	1.000	0.213	0.610	0.849	0.213	0.588	0.763	0.648	0.648	0.489	0.641	0.641	0.393	0.909	0.648
**WMHV + RF**	0.227	0.677	0.922	0.227	0.342		1.000	0.213	0.302	0.890	0.742	0.453	0.922	0.588	0.435	0.635	0.620	0.849	0.636	0.635	0.890	0.445	0.610
**PCV + RF**	0.213	0.622	1.000	0.213	0.227	1.000		0.213	0.213	0.922	0.636	0.339	1.000	0.588	0.339	0.588	0.584	0.724	0.588	0.588	0.742	0.317	0.504
**CLUSTERS + RF**	0.922	0.584	0.307	0.922	0.922	0.213	0.213		1.000	0.213	0.453	0.784	0.213	0.339	0.648	0.627	0.620	0.489	0.621	0.620	0.375	0.769	0.588
**DISTANCE + RF**	0.922	0.616	0.325	0.922	1.000	0.302	0.213	1.000		0.213	0.572	0.742	0.213	0.504	0.636	0.620	0.620	0.445	0.616	0.616	0.355	0.849	0.616
**WMHV + PCV**	0.213	0.435	0.818	0.213	0.213	0.890	0.922	0.213	0.213		0.453	0.227	1.000	0.504	0.302	0.471	0.445	0.584	0.471	0.453	0.620	0.227	0.393
**WMHV + CLUSTERS**	0.523	0.922	0.724	0.317	0.610	0.742	0.636	0.453	0.572	0.453		0.761	0.584	0.922	0.849	0.922	0.922	1.000	0.922	0.922	0.922	0.742	0.922
**WMHV + DISTANCE**	0.688	0.801	0.471	0.636	0.849	0.453	0.339	0.784	0.742	0.227	0.761		0.227	0.742	1.000	0.899	0.896	0.620	0.890	0.868	0.556	0.922	0.896
**WMHV + PCV + RF**	0.213	0.588	0.922	0.213	0.213	0.922	1.000	0.213	0.213	1.000	0.584	0.227		0.489	0.227	0.523	0.489	0.622	0.556	0.523	0.621	0.227	0.435
**WMHV + CLUSTERS + RF**	0.393	0.922	0.742	0.445	0.588	0.588	0.588	0.339	0.504	0.504	0.922	0.742	0.489		0.784	0.922	0.922	1.000	0.922	0.922	0.922	0.724	0.922
**WMHV + DISTANCE + RF**	0.620	0.868	0.504	0.616	0.763	0.435	0.339	0.648	0.636	0.302	0.849	1.000	0.227	0.784		0.922	0.922	0.742	0.922	0.922	0.616	1.000	0.922
**RWMHV**	0.588	0.922	0.620	0.588	0.648	0.635	0.588	0.627	0.620	0.471	0.922	0.899	0.523	0.922	0.922		0.899	0.861	0.922	0.922	0.763	0.884	0.922
**RWMHV + RF**	0.588	0.922	0.620	0.588	0.648	0.620	0.584	0.620	0.620	0.445	0.922	0.896	0.489	0.922	0.922	0.899		0.849	0.922	0.922	0.724	0.861	0.922
**RWMHV + PCV**	0.469	1.000	0.792	0.423	0.489	0.849	0.724	0.489	0.445	0.584	1.000	0.620	0.622	1.000	0.742	0.861	0.849		0.861	0.861	1.000	0.604	0.868
**RWMHV + CLUSTERS**	0.588	0.922	0.620	0.584	0.641	0.636	0.588	0.621	0.616	0.471	0.922	0.890	0.556	0.922	0.922	0.922	0.922	0.861		0.922	0.792	0.884	0.922
**RWMHV + DISTANCE**	0.588	0.922	0.620	0.588	0.641	0.635	0.588	0.620	0.616	0.453	0.922	0.868	0.523	0.922	0.922	0.922	0.922	0.861	0.922		0.792	0.868	0.922
**RWMHV + PCV + RF**	0.342	0.922	0.899	0.342	0.393	0.890	0.742	0.375	0.355	0.620	0.922	0.556	0.621	0.922	0.616	0.763	0.724	1.000	0.792	0.792		0.523	0.742
**RWMHV + CLUSTERS + RF**	0.661	0.788	0.453	0.644	0.909	0.445	0.317	0.769	0.849	0.227	0.742	0.922	0.227	0.724	1.000	0.884	0.861	0.604	0.884	0.868	0.523		0.784
**RWMHV + DISTANCE + RF**	0.584	0.922	0.610	0.556	0.648	0.610	0.504	0.588	0.616	0.393	0.922	0.896	0.435	0.922	0.922	0.922	0.922	0.868	0.922	0.922	0.742	0.784	
